# Managing an Agent's Self-Presentational Strategies During an Interaction

**DOI:** 10.3389/frobt.2019.00093

**Published:** 2019-09-24

**Authors:** Beatrice Biancardi, Maurizio Mancini, Paul Lerner, Catherine Pelachaud

**Affiliations:** ^1^CNRS-ISIR, Sorbonne University, Paris, France; ^2^School of Computer Science and Information Technology, University College Cork, Cork, Ireland; ^3^CNRS-LIMSI, Université Paris-Saclay, Paris, France

**Keywords:** embodied conversational agents, warmth, competence, human-agent interaction, impression management, non-verbal behavior

## Abstract

In this paper we present a computational model for managing the impressions of warmth and competence (the two fundamental dimensions of social cognition) of an Embodied Conversational Agent (ECA) while interacting with a human. The ECA can choose among four different self-presentational strategies eliciting different impressions of warmth and/or competence in the user, through its verbal and non-verbal behavior. The choice of the non-verbal behaviors displayed by the ECA relies on our previous studies. In our first study, we annotated videos of human-human natural interactions of an expert on a given topic talking to a novice, in order to find associations between the warmth and competence elicited by the expert's non-verbal behaviors (such as type of gestures, arms rest poses, smiling). In a second study, we investigated whether the most relevant non-verbal cues found in the previous study were perceived in the same way when displayed by an ECA. The computational learning model presented in this paper aims to learn in real-time the best strategy (i.e., the degree of warmth and/or competence to display) for the ECA, that is, the one which maximizes user's engagement during the interaction. We also present an evaluation study, aiming to investigate our model in a real context. In the experimental scenario, the ECA plays the role of a museum guide introducing an exposition about video games. We collected data from 75 visitors of a science museum. The ECA was displayed in human dimension on a big screen in front of the participant, with a Kinect on the top. During the interaction, the ECA could adopt one of 4 self-presentational strategies during the whole interaction, or it could select one strategy randomly for each speaking turn, or it could use a reinforcement learning algorithm to choose the strategy having the highest reward (i.e., user's engagement) after each speaking turn.

## 1. Introduction and Motivation

During the last decades, anthropomorphic interfaces, such as humanoid robots and virtual characters, have been increasingly deployed in several roles, such as pedagogical assistants, companion, trainers. When conceiving Embodied Conversational Agents (ECAs), which are anthropomorphic virtual characters capable of interacting with users using verbal and non-verbal behavior (for more details, see Cassell, 2000), it is very important to take into account how users perceive them during the course of the interaction. Virtual agents ought to be endowed with the capability of maintaining engaging interactions with users (Sidner and Dzikovska, 2005). This would make it easier for a virtual guide to transmit information, would ensure change behavior for a virtual coach, would create rapport with a virtual companion. Like in human-human interactions, the first moments of an interaction with a virtual character are critical since users form impressions about them, that can affect the rest of the interaction, in terms of engagement and willingness to continue it (Cafaro et al., 2016).

During the first moments of a new encounter, people automatically collect information to infer the intentions of the others (also called “warmth” dimension Fiske et al., 2007), that is, how the others seem friendly, social, moral, as well as the consequent ability to enact those intentions (called “competence” dimension Fiske et al., 2007), that is, how the others seem intelligent, competent, skillful. People are quite accurate at forming this kind of impressions, by collecting and integrating information from others' appearance and behaviors. This process, defined by Goffman and his colleagues as *impression formation*, is naturally coupled with *impression management*, that is, the attempt to control the impressions that one gives to the others (Goffman et al., 1978). Impression management concerns, among other, dressing and hairstyle, the choice of the moment when smiling, as well as behaviors such as body orientation, posture, etc. People adopt verbal and non-verbal self-presentational strategies in order to elicit in the other a specific impression. According to the context and the goal, one can choose a strategy to convince a target other that he is likable or competent for example (Jones and Pittman, 1982).

Non-verbal behaviors play an important role in these processes (Goffman et al., 1978; Judd et al., 2005). If we want to investigate the effects of these behaviors on the interaction, this could be difficult since we cannot have full control of them in a spontaneous interaction between humans. We can exploit ECAs, which allow us to fully manage their behaviors, to investigate the effect of non-verbal behaviors on the interaction.

In the work presented in this paper, we manage agent's behaviors. To choose the set of possible behaviors for the agent to display, we previously started from the analysis of human-human interaction, in order to identify non-behavioral cues eliciting different impressions of warmth and competence (Biancardi et al., 2017). We then implemented them into an ECA in order to investigate how these cues are perceived when displayed by a virtual character instead of a human (Biancardi et al., 2018). Starting from these findings, we now focus on two main questions:

- What is the impact of these behaviors on a real interaction between an ECA and a human?
- How can an ECA manage its behaviors in order to engage the user, and so to improve the quality of the interaction?

To address them, we have developed a model to manage the impressions generated by an ECA on the user, by endowing it with the capability of adapting its behaviors, and the strategies that drive them, according to user's reactions. The goal of the agent is to maximize user's engagement during the interaction. If the user is engaged, it is more probable for her to have a longer interaction and to appreciate it.

In the following sections, we will describe the dimensions studied in this work in section 2 and the related work in section 3, we will present the architecture of our system in section 4 and the evaluation study of the system in section 5. We will finally discuss the results in section 6 and the limitations and possible improvements of our system in section 7.

## 2. Background

In this section we provide definitions and related theories about the psychological dimensions that are investigated in our research: the two fundamental dimensions of social cognition, that is, Warmth and Competence (W&C), and Engagement.

### 2.1. Warmth and Competence

Several authors investigated the fundamental dimensions of social cognition, that is, those characteristics of the others that are processed from the initial moments of an interaction.

These authors converged, even if adopting different terminology, to two main dimensions (Abele and Wojciszke, 2013). The first includes traits like friendliness, morality, sociability, trustworthiness, and it is commonly labeled as warmth. The second one includes traits like agency, efficacy, intelligence, and it is commonly labeled as competence. In the current work we refer to competence as cognitive competence (knowledge, abstract intelligence and experience).

We can already find W&C in Asch's research (Asch, 1946). He was the first who intuited the centrality of W&C in impression formation. Later, Rosenberg et al. distinguished intellectual good/bad traits (such as intelligent, skillful, determined, foolish, unintelligent, irresponsible) and social good/bad traits (such as sociable, honest, warm, unsociable, cold, unhappy) as the main dimensions of person's judgements (Rosenberg et al., 1968). Wojciszke et al. showed that W&C account for almost 82% of the variance in global impressions of well-known others: when people interpret behaviors or their impressions of others, W&C form basic dimensions that almost entirely account for how people characterize others (Wojciszke et al., 1998).

According to the evolutionary explanation given by Fiske et al. warmth is judged before competence, as others' intentions matter more to survival whether the other can act on those goals (Fiske et al., 2007). Primacy of warmth is supported by a large evidence (Willis and Todorov, 2006; Wojciszke and Abele, 2008). In Wojciszke and Abele (2008) participants were asked to list the most important personality traits: they listed significantly more warmth traits than competence traits, and the five most frequently listed traits were warmth-related. Moreover, evaluations based on warmth information were strong and stable, while those based on competence information were weak and dependent on accompanying warmth information. Finally, cognitive performance is better for warmth than for competence. For example, in rapidly judging faces at 100 ms exposure times, social perceivers judged trustworthiness (as a warmth trait Fiske et al., 2007) most reliably, followed by competence (Willis and Todorov, 2006).

Whether the previous authors investigated W&C at a person-perception level, Fiske et al. with their Stereotype Content Model (Fiske et al., 2002), showed the role of W&C in group stereotypes. Groups' warmth is judged according to their level of competition with the in-group, while competence depends on the group status. Different levels of W&C elicit unique emotional (admiration, contempt, envy, and pity; Fiske et al., 2002) and behavioral responses (active and passive, facilitative and harmful; Cuddy et al., 2008).

Another topic of interest concerning W&C is the relationship between the judgements about them. According to Rosenberg et al. they are positively correlated, that is, a *halo effect* occurs (Rosenberg et al., 1968). This effect led people who were given information about only one dimension (warmth or competence), to make judgements about the other (non-described) dimension toward the same direction of the described one.

Yzerbyt et al. showed evidence for an opposite effect instead, called *compensation effect* (Yzerbyt et al., 2008). This effect also occurred in Judd et al. experiments, where they asked to compare two targets. Some participants received information about the competence of the two targets (high in one target and low in the other one), while other participants received information about the warmth of the two targets (again, high in one target and low in the other one). Judgements about the manipulated dimension (competence for some participants, warmth for the others) corresponded to the given information, while for the non-manipulated dimension they went toward the opposite direction of those about the manipulated dimension (Judd et al., 2005).

More recent studies showed the occurrence of compensation effect also in absence of any explicit comparative context, that is without evoking any explicitly comparison to another target. Kervyn et al. called it *amplification effect* (Kervyn et al., 2016).

#### 2.1.1. Behavioral Cues of Warmth and Competence

While most of the studies described above used written descriptions of traits and situations as cues of W&C (e.g., “X helped a blind woman to cross the street,” “X wrote a little computer program that solved a tough calculus integration problem”), other works focused on non-verbal cues conveying these dimensions.

Previous research in human-human interaction showed an important effect of smiling on warmth (Bayes, 1972; Cuddy et al., 2008), as well as the presence of immediacy cues that indicate positive interest or engagement (e.g., leaning forward, nodding, orienting the body toward the other), touching and postural openness, and mirroring (i.e., copying the non-verbal behaviors of the interaction partner). Leaning backwards, orientating the body away from the other, tense and intrusive hand gestures (e.g., pointing) are related to impressions of low warmth (Cuddy et al., 2008).

Non-verbal behaviors eliciting competence are more related to dominance and power, such as expansive (i.e., taking up more space) and open (i.e., keeping limbs open and not touching the torso) postures. People who express high-power or assertive non-verbal behaviors are perceived as more skillful, capable, and competent than people expressing low-power or passive non-verbal behaviors (Cuddy et al., 2008). Hand gestures have been found to influence competence perception too, in particular, ideationals (i.e., gestures related to the semantic content of the speech) and object-adaptors resulted in higher judgements of competence, while self-adaptors resulted in lower ones (Maricchiolo et al., 2009).

#### 2.1.2. Self-Presentational Strategies

Jones and Pittman argued that people can use different verbal and non-verbal behavioral techniques to create the impressions they desire in their interlocutor (Jones and Pittman, 1982). The authors proposed a taxonomy of these techniques, that they called self-presentational strategies. We illustrate here 4 of their strategies that can be associated to different levels of W&C. We did not consider the 5th strategy of the taxonomy, called *Exemplification*. This strategy is used when people want to be perceived as self-sacrificing and to gain the attribution of dedication from others, thus it is not related neither to warmth nor to competence. Concerning the other 4 strategies, two of them focus on one dimension at a time, the other two focus on both dimensions by giving them opposite values:

- *Ingratiation*: its goal is to get the other person to like you and attribute positive interpersonal qualities (e.g., warmth and kindness). The person selecting this strategy has the goal to elicit impressions of high warmth, without considering its level of competence.
- *Supplication*: it occurs when individuals present their weaknesses or deficiencies to receive compassion and assistance from others. The person selecting this strategy has the goal to elicit impressions of high warmth and low competence.
- *Self-promotion*: it occurs when individuals call attention to their accomplishments to be perceived as capable by observers. The person selecting this strategy has the goal to elicit impressions of high competence, without considering its level of warmth.
- *Intimidation*: it is defined as the attempt to project its own power or ability to punish to be viewed as dangerous and powerful. In the context of our research, we interpret this strategy in a smoother way, as the goal to elicit impressions of low warmth and high competence.

### 2.2. Engagement in Human-Agent Interaction

An important aspect of human-agent interaction is engagement which ensures the interaction to move forwards. Despite of being a major theme of research and a universal goal in Human-Computer Interaction (HCI), engagement is a difficult concept to define (102 different definitions of engagement exist according to Doherty and Doherty review Doherty and Doherty, 2018), due to its multidimensional nature and the difficulty to measure it.

A detailed summary of engagement definitions in human-agent interaction is provided in Glas and Pelachaud (2015a). Among others, it can be defined as “the value that a participant in an interaction attributes to the goal of being together with the other participant(s) and of continuing the interaction” (Poggi, 2007), and as “the process by which participants involved in an interaction start, maintain and terminate an interaction” (Sidner and Dzikovska, 2005; Corrigan et al., 2016).

Engagement is not measured from single cues, but rather from several cues that arise over a certain time window (Peters et al., 2005). Engagement can be defined by high-level behavior like, synchrony—which is the temporal coordination during social interactions; mimicry—which is the automatic tendency to imitate others; feedback—which can indicate whether the communication is successful or not. Similarly, engagement can also be defined by low-level behavior like eye gaze - providing feedback and showing interest; head movements - nods (in agreement, disagreement, in between); gestures—to greet, to take turns; postures—body orientation, lean; facial expressions. Clavel et al. provided a review on methodologies for assessing user engagement in human-agent interaction (Clavel et al., 2016).

In the work presented in this Chapter we used low-level signals, such as facial Action Units activation, trunk and head rotation, to measure engagement. The engagement detection model is described in section 4.1.

## 3. Related Work

Some works already exist that included W&C dimensions in ECAs. Nguyen et al. analyzed gestures, use of space and gaze behaviors in videos of actors performing different degrees of W&C (Nguyen et al., 2015). They applied an iterative methodology which included theory from theater, animation and psychology, expert reviews, user testing and feedback, in order to extract a set of rules to be encoded in a virtual agent. They then asked participants to rate W&C of an agent behaving by following these rules. Bergmann et al. found that human-like vs. robot-like appearance positively affects impressions of warmth, while the presence of co-speech gestures increases competence judgements (Bergmann et al., 2012).

The goal of our current work is to model W&C dimensions in order to obtain an engaging ECA, by following the idea that a more engaging agent is likely to form a positive impression and be accepted by the user, thus promoting further interactions (Bergmann et al., 2012; Cafaro et al., 2017). Several authors attempted to design engaging virtual agents, by focusing on the use of feedback and backchannels (Truong et al., 2010), by adopting politeness strategies (Glas and Pelachaud, 2015b), or by investigating the role of verbal alignment for improving user's engagement (Campano et al., 2015). Other studies focused on how to improve user's engagement by adapting social agents (mainly robots) behaviors, using reinforcement learning (RL) methods. These works incorporate user's social signals to measure user's engagement and exploit it as the reward of the RL algorithm. For example, Ritschel et al. computed user's engagement as a reward, with the goal to adapt robot's personality expressed by linguistic style (Ritschel et al., 2017). Gordon et al. exploited facial expressions to measure child's engagement in order to adapt a robot's behaviors (Gordon et al., 2016), while Liu et al. exploited user's physiological signals (Liu et al., 2008).

### 3.1. Our Previous Work

In our previous research, we investigated the associations between non-verbal cues and W&C impressions in human-human interaction (Biancardi et al., 2017). To do that, we annotated videos form NoXi dataset (Cafaro et al., 2017), a corpus of spontaneous interactions involving an expert and a novice discussing about a given topic (e.g., sports, videogames, travels, music, etc.). We annotated the type of gesture, the type of arms rest poses, head movements and smiling, as well as the perceived W&C of the expert. We found a negative association with warmth and competence for some arms rest poses like arms crossed. We also found that the presence of gestures was positively associated with both W&C, in particular the presence of beat gestures (rhythmic gestures not related to the speech content) for both W&C and ideationals for warmth. In addition, when gestures were performed with a smile, warmth judgements increased. A *compensation effect* was found for smiling: warmth judgements were positively related to the presence of smiles, while competence judgements were negatively related to it.

With respect to the works cited at the beginning of the section, we considered more behaviors than only co-speech gestures, in particular the position of the arms when not performing gestures. In addition, we analyzed W&C elicited by non-verbal behaviors performed during natural interactions, instead of behaviors performed by actors.

We then continued our research by questioning how these cues are perceived when displayed by an ECA (Biancardi et al., 2018). To do that, we manipulated in an ECA the most interesting findings from the previous study and asked people to rate videos of the agent displaying different combinations of these manipulations. We found an effect of type of gesture on W&C judgements. In particular, W&C ratings were higher when the agent displayed ideationals than compared to when it displayed beats. In addition, this effect occurred for warmth judgements only when the frequency of gestures was high rather than low.

Our previous works did not investigate W&C impressions in an interaction, where participants are no more passive observers but active agents. The work presented in this paper aims to improve the previous ones, by starting from their findings and focusing on two main questions:

- What is the impact of these behaviors on a real interaction between an ECA and a human?
- How can an ECA manage its behaviors in order to engage the user, and so to improve the quality of the interaction?

We conceived an interaction scenario where the agent manages the impressions of W&C it gives by adopting one of the 4 self-presentational strategies described in section 2.1.2. We exploited the results of our previous works in order to define the non-verbal behaviors associated to each strategy, while we relied on literature to select the verbal behavior for each strategy (see section 5).

In order to make the agent learn how to manage its impressions, that is, to adapt its behavior in real-time to user's engagement level, we adopt a reinforcement learning (RL) approach rather than supervised learning techniques. Since the ECA's behavioral adaptation has the goal to maximize user's engagement, we use this variable as reward in the RL algorithm. The action space, that is, the set of possible choices of the agent, concerns different behavioral strategies, eliciting impressions of different levels of W&C.

Differently from the existing works described above, the system presented in this paper is the first one using behaviors eliciting different W&C impressions as variables in a RL algorithm for ECAs.

To do this aim, we implemented a system architecture that is described in more details in the following section.

## 4. System Architecture

We conceived a system architecture to enable the interaction between an ECA and a user. To do that, we implemented software modules to capture user's behavior (speech, facial expressions, head and torso orientation), analyse/interpret it (e.g., detect the user's level of engagement) and decide what the ECA should say and how (i.e., the non-verbal behaviors accompanying speech). The ECA's speech and behavior are decided not only based on the detected user's level of engagement but also by taking into account the ECA's self-presentational intention. That is, the ECA has the goal of communicating a given level of W&C that will influence the choice of the verbal and non-verbal signals to be produced.

**Figure 1:**
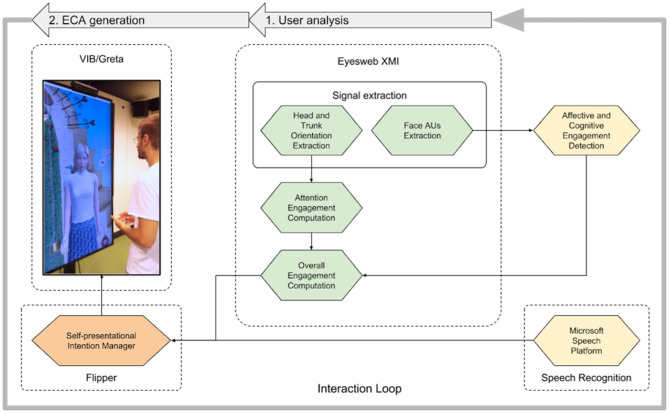
System architecture: user non-verbal and verbal signals are extracted by EyesWeb and the Microsoft Speech Platform, respectively; user's overall engagement, computed by EyesWeb, is provided to the Self-presentational Intention Manager that decides the verbal and non-verbal signals to be produced by VIB/Greta. (The person in this image agrees for publication).

Figure 1 illustrates the system we designed and implemented. We can distinguish 2 main parts:

1. *User analysis*–We exploit the EyesWeb platform (Camurri et al., 2004), that extracts in real-time: (1) user's non-verbal signals (i.e., torso and head orientation), starting from the Kinect depth camera skeleton data; (2) user's face Action Units (AUs), by running the OpenFace framework (Baltrušaitis et al., 2016); (3) user's speech, by executing the Microsoft Speech Platform^1^. After that, as illustrated in section 4.1, EyesWeb computes the user's overall engagement.
2. *ECA generation*–Agent's behavior generation is performed by VIB/Greta, a software platform supporting the creation of socio-emotional embodied conversational agents (Pecune et al., 2014). For the presented work, we implemented a self-presentational intention manager using Flipper (van Waterschoot et al., 2018) to process the detected user's overall engagement and speech and to choose the verbal and non-verbal signals the ECA has to perform in the next speaking turn, according to a reinforcement learning algorithm. The self-presentational intention manager also includes a Natural Language Processing (NLP) module for user's speech interpretation. As explained in section 4.2, Flipper selects the proper communicative intention of the ECA while VIB/Greta generates the ECA animation consisting of gestures, facial expressions and gaze, in sync with speech.

### 4.1. Overall Engagement Detection

As mentioned earlier in the paper, in this work we aim at endowing ECAs with the capability of adapting their behavior according to the user's reactions. In particular, we focus on the user's level of engagement. So, we now present our computational model of user's engagement based on the works of Corrigan et al. (2016) and Sidner and Dzikovska (2005). In our model, user's engagement can be expressed at three different levels, corresponding to different types of non-verbal signals:

- *Attention engagement*–Engagement can be expressed by continuously gazing at relevant objects/persons during the interaction. The more a person continuously focuses her attention on a relevant object/person, the more engaged she is (Sidner and Dzikovska, 2005).
- *Cognitive engagement*–(Corrigan et al., 2016) claims that “frowning may indicate effortful processing suggesting high levels of cognitive engagement.” The same work also refers to signals such as “looking for a brief interval outside the scene” as indicators of cognitive engagement.
- *Affective engagement*–Smiling could indicate that a person is enjoying the interaction, while some postures (e.g., crossed arms, hands in pockets) or posture shifts can indicate a lack of engagement.

The *Affective and Cognitive Engagement Detection* module is based on a Long Short-Term Memory (LSTM) prediction model using Recurrent Neural Networks implemented with the Keras toolkit and TensorFlow. More details about this model can be found in Dermouche and Pelachaud (2018). The prediction model takes as input the user's face AUs during the last second, and predicts the user's affective and cognitive engagement: for example, when non-verbal signals like frowning or smiling are extracted, the affective and cognitive engagement increases.

The *Attention Engagement Computation* module is implemented in EyesWeb as a set of rules. It takes as input the user's head and torso orientation and computes the user's attention engagement: for example, if the user is facing the ECA (with both her head and torso) then the attention engagement increases.

Finally, affective, cognitive and attention engagement are summed up by the *Overall User Engagement Computation* module.

Overall user's engagement is computed continuously at 10 Hz during every speaking turn, starting when the agent starts to pronounce its question for the user and ending when the user stops replying to the agent (or, if the user does not respond, until a 1,500 ms of continuous silence is detected). After the end of the speaking turn, the overall mean engagement is sent from EyesWeb to the Self-presentational Intention Manager, described in the following section, that will plan the verbal and non-verbal behavior the ECA will produce in the next speaking turn.

**Figure 2:**
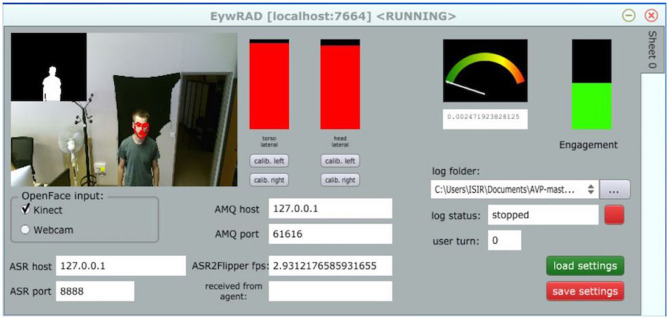
The user analysis interface implemented in EyesWeb. On the left, user's silhouette is extracted from Kinect's depth data. The two red bars in the middle indicate that the user is looking at the screen, with both her trunk (left bar) and head (right bar). Audio intensity is low (volume meter on the right), that is, the user is not speaking. Overall engagement level is represented by the green bar on the right (The person in this image agrees for publication).

Figure 2 depicts the user analysis interface, developed in EyesWeb.

### 4.2. Self-Presentational Intention Manager

User's speech and overall engagement are sent to the Self-presentational Intention Manager implemented in the Dialog Manager Flipper, an open-source engine for pragmatic yet robust interaction management for ECAs (van Waterschoot et al., 2018).

The Dialog Manager Flipper is based on two main components described in XML: the *information state* and the *declarative templates*. The information state stores interaction-related information and data in a hierarchical tree-based structure. Declarative templates can be grouped and organized in different files according to their related functionality (van Waterschoot et al., 2018). Each template consists of:

- *preconditions*: sets of rules that describe when a template should be executed;
- *effects*: associated updates to the information state.

So, for example, we defined a template whose precondition is that the user's overall engagement value has been computed by EyesWeb (see section 4.1) and the effect is that the expected reward of the current self-presentational intention has to be updated depending on the engagement value (see section 4.2.1).

Flipper has been also exploited to implement a dialogue manager based on NLP, aiming at interpreting user's speech to select the ECA's next self-presentational intention. Since the generation of a realistic and complex dialogue is not the main focus of our work, the agent takes into account only the polarity of user's answers rather than the semantic content of user's speech. For example, the agent can ask whether or not the user wants a more detailed explanation about a topic: if the user's answer is positive, then the agent will talk about it in more detail, or will move to another topic in case of a negative answer (see section 5.5).

#### 4.2.1. Self-Presentational Intention Selection

During its interaction with the user, the ECA has the goal of selecting its self-presentational intention (e.g., to communicate verbally and non-verbally a given utterance with high warmth and low competence). The ECA will choose its intention among a given set of possible utterances depending on the user's overall engagement value: for example, if the last self-presentational intention had the effect of decreasing the detected user's engagement, then the ECA will select a different intention for the next speaking turn, that is it will select an utterance associated with a different value of warmth and of competence; conversely, if the last intention increased user's engagement, that intention will be maintained.

This problem can be seen as a *multi-armed bandit problem* (Katehakis and Veinott Jr, 1987), which models agents evolving in an environment where they can perform several actions, each action being more or less rewarding for them.

In our case, the actions that the ECA can perform are the verbal and non-verbal behaviors corresponding to the self-presentational intention the ECA aims to communicate, and they are selected by the Formula 1. The environment is the interaction with the user, while the state space is the set of the topics discussed at each speaking turn, and it is defined by the Dialog Manager. That is, the choice of the action does not change the state (i.e., the topic discussed during the actual speaking turn), but rather it acts on how this topic is realized by verbal and non-verbal behavior.

In order to maximize user's engagement during the interaction, the ECA will, at the beginning, explore the environment (i.e., by randomly choosing an initial self-presentational intention) and then exploit its knowledge (i.e., user's engagement) to find the most rewarding self-presentational intention.

To do that, we choose to exploit the ϵ-decreasing learning approach: the exploration rate ϵ continuously decreases in time. In this way, the ECA starts the interaction with the user by exploring the environment without taking into account knowledge (i.e., user's engagement) and finishes it by exploiting its knowledge only (i.e., without performing any further environment exploration). That is, the ECA explores with probability ϵ, and exploits knowledge with probability 1 − ϵ.

The ECA updates its knowledge through a table where it iteratively approximates the expected reward *Q*(*int*) of a self-presentation intention *int*. This is done using the formula below:

(1) $$\begin{array}{l} Q{\left(int\right)}_{t+1}\leftarrow \left(1-\alpha \right)\times Q{\left(int\right)}_{t}+\alpha \times e_{t} \end{array}$$

where:

- *Q*(*int*) is the expected value of the intention, *int* ∈ [*ingratiation, supplication, self-promotion, intimidation*];
- α is the learning rate, set at 0.5, a very high number compared to other works (e.g., in Burda et al., 2018 it was set to 0.0001). This is because the ECA needs to learn quickly (i.e., in few dialogue steps) the self-presentational intention to use;
- *e* is the overall engagement score, that is the reward for the ECA.

## 5. Evaluation Study

We now present the evaluation study we conceived to investigate whether or not an ECA endowed with the architecture described in the previous section, that is, able to manage its impressions of W&C according to user's engagement, could affect user-agent interaction. In the study, we compared different conditions where the ECA could interact with the user by adapting or not its behaviors.

We created a scenario where the virtual agent, called Alice, plays the role of a virtual guide of a museum. The experiment took place in the Carrefour Numerique, an area of the Cité des sciences et de l'industrie in Paris, one of the largest sciences museums in Europe.

### 5.1. Independent Variables

The independent variable manipulated in this study concerns agent's **Strategy**, that is, how the agent manages its behaviors to influence user's perception of its W&C.

For each speaking turn, the agent plays one out of 4 self-presentational techniques presented in section 2.1.2, inspired from Jones & Pittman's taxonomy (Jones and Pittman, 1982), in order to appear more or less warmth and/or competent. According to the different **Strategy** conditions, the agent can select one of the 4 self-presentational techniques at the beginning and display it during the whole interaction, or select one of the 4 at each speaking turn, either randomly or by using our self-presentational intention model based on user's overall engagement detection.

**Figure 3:**
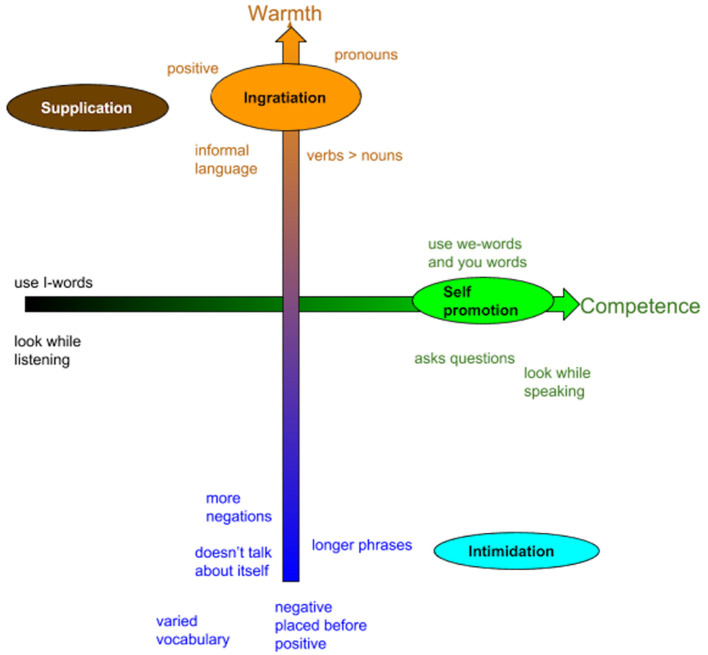
Use of pronouns, verbs, type of language, and other verbal behaviors associated to each self-presentational technique, inspired from Pennebaker (2011) and Callejas et al. (2014).

These 4 self-presentational techniques are realized by the agent through its non-verbal and verbal behavior. The choice of its non-verbal behavior is based on our previous studies described in section 3.1. The verbal behavior characterizing the different strategies is inspired by the works of Pennebaker (2011) and Callejas et al. (2014). According to their findings, we manipulated the use of *you*- and *we*- pronouns, the level of formality of the language, the length of the sentences. For example, sentences aiming at eliciting high warmth contain more pronouns, less synonyms, more informal language, so that the phrases are more casual and give the impression to be less meditated; more verbs rather than nouns, and positive contents are predominant. Sentences aiming at eliciting low warmth contain more negations, longer phrases, formal language, and do not refer to the speaker. Sentences aiming at eliciting high competence contain high rates of we- and you-words, and I-words at low rates. Figure 3 shows the use of verbal behavior according to each self-presentational technique, while Table 1 shows an example of a speaking turn for each of the 4 techniques.

**Table 1:** An example of 4 different sentences for the same speaking turn (the agent introduces the videogames exhibition), according to the 4 different self-presentational techniques.

**Strategy**	**Translated sentence**	**Original sentence**
**INGR**	“You can test some games, if you wanna.”	*Tu vas pouvoir tester des jeux si tu veux*.
**SUPP**	“I dunno about the other exhibits of the museum, but here you can test some games, it's cool!”	*J'connais pas les autres expo du musée, mais ici on peut tester des jeux, c'est trop bien !*
**SELF**	“In this exhibition, you can test some videogames.”	*Dans cette expo tu va pouvoir tester des jeux-vidéos*.
**INTIM**	“In this exhibition, you can try out some games on different platforms.”	*Dans cette exposition tu peux essayer des jeux sur différents supports*.

*The original sentences in French are provided*.

The independent variable **Strategy** has 6 levels: the first 4 levels are static conditions, where one self-presentational technique is chosen at the beginning of the interaction and does not change; in the last 2 levels the self-presentational technique is chosen at each speaking turn. They are:

- **INGR:** when the agent selects the Ingratiation self-presentational technique from the beginning to the end of the interaction, without considering user's reactions;
- **SUPP:** when the agent selects the Supplication self-presentational technique from the beginning to the end of the interaction, without considering user's reactions;
- **SELF:** when the agent selects the Self-promotion self-presentational technique from the beginning to the end of the interaction, without considering user's reactions;
- **INTIM:** when the agent selects the Intimidation self-presentational technique from the beginning to the end of the interaction, without considering user's reactions;
- **RAND:** it consists in selecting one of the 4 self-presentational techniques, randomly, at each speaking turn, **without considering user's reactions**;
- **IMPR:** it consists in selecting one of the 4 self-presentational techniques, at each speaking turn, **by using our self-presentational intention model** based on user's overall engagement detection (see section 4.1).

According to the **Strategy** level, the self-presentational intention selection module of the Dialog Manager Flipper (see section 4.2.1) will apply (or not) the reinforcement learning formula 1 to update the action (i.e., the following self-presentational intention) of the agent.

### 5.2. NARS

Before the interaction, we collected information about users' attitudes and prejudices toward virtual characters. We used a slightly adapted version of the Negative Attitudes toward Robots Scale (Nomura et al., 2006). This questionnaire measures people's negative attitudes toward situations and interactions with robots, toward the social influence of robots, and toward emotions in interaction with robots. We selected the most relevant questions according to our context and adapted the questions by referring to virtual characters instead of robots. Participants gave their rating on a 5-points Likert scale, from 1 = “I completely disagree” to 5 = “I completely agree.” The items of the questionnaires (translated in English) are available in Table 2.

**Table 2:** Items of the NARS questionnaire, adapted from Nomura et al. (2006).

**Items**
1. I would feel uneasy if virtual characters had emotions.
2. I would feel relaxed talking with virtual characters.
3. I feel comforted being with virtual characters that have emotions.
4. The word “virtual character” means nothing to me.
5. I would hate the idea that virtual characters were making judgements about things.
6. I would feel very nervous just standing in front of a virtual character.
7. I would feel paranoid talking with a virtual character.
8. I am concerned that virtual characters would be a bad influence on children.

### 5.3. Dependent Variables

The dependent variables were measured during and after the interaction with the virtual character. During the interaction, if the participant agreed in the consent form, we recorded the user's speech audio, in order to measure user's cues of engagement from his verbal behavior. After the interaction we asked the participants to rate the agent's W&C, and their overall satisfaction of the interaction.

#### 5.3.1. Verbal Cues of Engagement

For people who agreed with audio recording of the experiment, we collected quantitative information about their answers, in particular:

- The polarity of the answer to Topic1_question (see section 5.5);
- The polarity of the answer to Topic2_question (see section 5.5);
- The number of any verbal feedback produced by the user during a speaking turn.

#### 5.3.2. Self-Report Assessment

After the interaction, the participants filled in a final questionnaire, divided in several parts. In particular we measured:

- User's perception of agent's warmth (**w**) and competence (**c**): we presented a list of adjectives referring to W&C and asked participants to indicate their agreement on a 5-points Likert scale (1 = “I completely disagree,” 5 = “I completely agree”) about how precisely each adjective described the character. The items were taken from Aragonés et al. (2015) scale, and were: *kind, pleasant, friendly, warm* for warmth, and *competent, effective, skilled, intelligent* for competence.
- User's perception of the interaction (**perception**): the second part of the questionnaire concerned a list of items adapted from those already used by Bickmore et al. (2011). They are shown in Table 3.

**Table 3:** Items of the questionnaire about user's perception of the interaction, adapted from Bickmore et al. (2011).

**Measure**	**Question**
**Satisfaction**	*I am satisfied with my interaction with Alice*.
**Continue**	*I would like to talk with Alice again*.
**Like**	*I liked Alice*.
**Learnfrom**	*I have learned something from Alice*.
**Exhib**	*Alice gave me want to visit the exhibition (if you haven't yet)*
**Rship**	*I would describe Alice as a complete stranger vs. a close friend*.
**Likeperson**	*I would describe Alice just as a computer vs. like a person*.

*Alice is the name of the virtual character*.

### 5.4. Hypotheses

The first experiment's goal was to demonstrate that the ECA's 4 self-presentational techniques during all the interaction are correctly perceived by users, for example, if users rate the agent in **INGR** condition as warm, and the agent in **INTIM** as cold and competent.

In particular, we hypothesize that:

- **H1ingr**: The agent in **INGR** condition will be perceived as **warm** by users;
- **H1supp**: The agent in **SUPP** condition will be perceived as **warm** and **not competent** by users;
- **H1self**: The agent in **SELF** condition will be perceived as **competent** by users;
- **H1intim**: The agent in **INTIM** condition will be perceived as **competent** and **not warm** by users.

Then, our main hypothesis is that the use of the self-presentational intention model based on user's overall engagement detection (i.e., when the virtual character adapts its behaviors) positively affects user's perception of the interaction. Thus, we hypothesize that:

- **H2a**: The scores of **perception** items are higher in **IMPR** condition compared to all the other conditions;
- **H2b**: The agent in **IMPR** condition influences how it is perceived in terms of W&C.

### 5.5. Protocol

**Figure 4:**
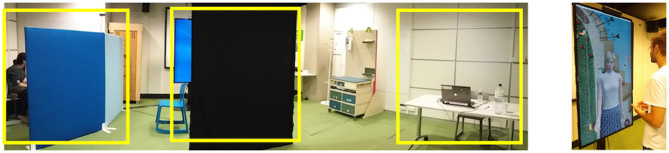
The experimenter room and an example of an interaction (the person in this image agrees for publication). In the yellow squares, on the left, the control place, in the middle the interaction place, and on the right the questionnaires space.

The experiment took place in a room of the Carrefour Numérique. As shown in Figure 4, the room was divided in three areas:

- The questionnaires place, including a desk with a laptop, and a chair;
- The interaction place, with a big screen displaying the virtual character, a Kinect 2 on the top of the screen and a black tent in front of the screen;
- The control station, separated by the rest of the room by 2 screens. This place included a desk with the computer controlling the system.

The experiment was completed in three phases:

1. Before the interaction begun, the participant sat at the questionnaires place, read and signed the consent form, and filled in a first questionnaire (see section 5.2), then moved to the interaction place, where the experimenter gave the last instructions (5 min);
2. During the interaction phase, the participant stayed right in front of the screen, between it and the black tent. He\she wore a headset and was free to interact with the virtual character as he \she wanted. During this phase, the experimenter stayed in the control place, behind the screens (3 min);
3. After the interaction, the participant came back to the questionnaires place and filled in the last questionnaires (see section 5.3.2). After that, the experimenter proceeded with the debriefing (5 min).

The interaction with the virtual character lasted about 3 min. It included 25–36 steps, according to user's answers. A step includes one or few sentences played by the virtual character and user's answer. If user did not reply in a certain interval of time, the agent started the following step. After each step, user's engagement was computed through our overall engagement detection model (see section 4.1).

The dialogue is divided into 4 main parts that were always played by the agent, no matter what answers the users gave:

1. Start interaction (8 steps);
2. Topic 1 (3 steps);
3. Topic 2 (4 steps);
4. End of the interaction (4 steps).

**Figure 5:**
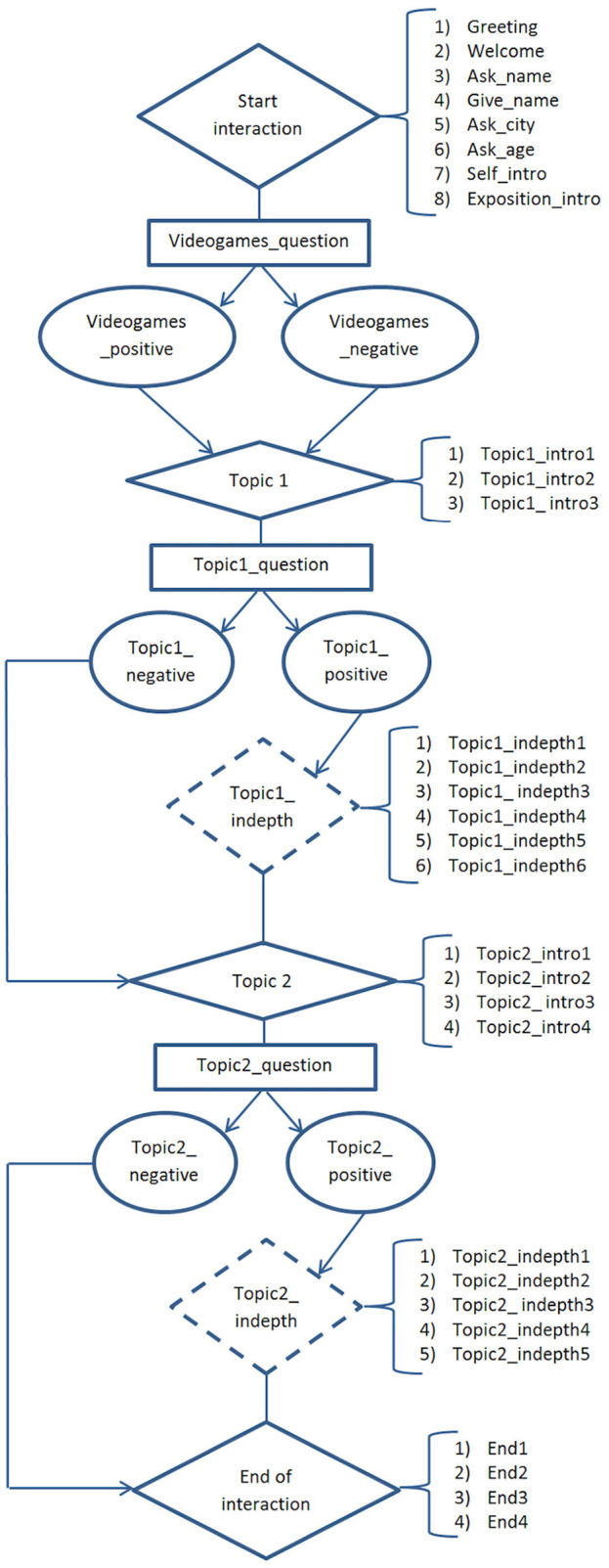
The dialogue flowchart. The diamond shapes represent the main parts that always occur during the dialogue, the rectangles represent questions, the rounds represent agent's reply to user's answer and the dotted shapes the optional parts. Where not specified, each shape represents one step of the dialogue.

At the end of parts 1, 2, and 3, the agent asked a question to the user. After parts 2 and 3, if the user gave a positive answer, the agent continued to talk about the same topic (6 steps for Topic 1, 5 steps for Topic 2), otherwise it skipped to the next part. The dialogue flowchart is shown in Figure 5.

### 5.6. Analysis and Results

We analyzed data from 75 participants, of which were 30 females and 2 preferred not to specify their gender. The majority of the participants were in the 18–25 or 36–45 age range, 5 of them were not native French speakers, and 72% of them had at least a Bachelor. Participants were almost equally distributed across the levels of the independent variable **Strategy** (12.5 ± 1 participants per each strategy).

Before conducting our analyses, we computed Cronbach's alphas and explored the distribution of data. Good reliability for **w** and **c** items was found (α = 0.9 and α = 0.8, respectively). We then used the mean of these items for our analyses. Since the distributions of this data satisfy assumptions for ANOVA, we run this type of test on them.

Nars scores got an acceptable score of reliability (α = 0.66), we therefore computed the means of these items in order to obtain one overall mean for each participant. We then divided participants into 2 groups, “high” and “low,” according to whether they obtained a score higher than the overall mean or not, respectively. Participants were almost equally distributed into the two groups (39 in the “high” group, 36 in the “low” group, almost equally distributed across the other variables, too).

#### 5.6.1. Warmth

A 4-way between-subjects ANOVA, including age, sex and Nars scores as factors, was first run in order to check for any effect of these variables. A main effect being found for Nars scores, we then conducted a 4 × 2 between-subjects ANOVA with **Strategy** and Nars as factors. The analysis revealed a main effect of **Strategy** [*F*_(5, 62)_ = 4.75, *p* = 0.000974, η^2^ = 0.26] and Nars [*F*_(1, 62)_ = 5.74, *p* = 0.02, η^2^ = 0.06]. Warmth ratings were higher from participants with a high Nars score (*M* = 3.74, *SD* = 0.77) than from those with a low Nars score (*M* = 3.33, *SD* = 0.92).

**Figure 6:**
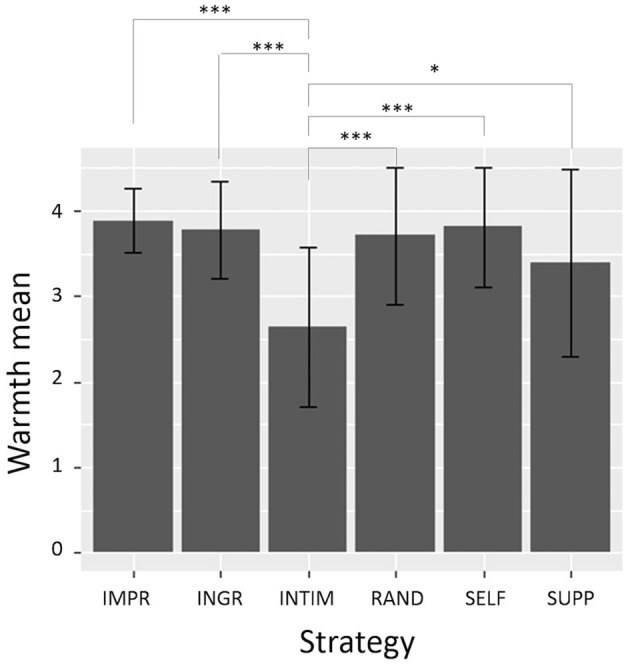
Mean and SD values of warmth ratings for each level of **Strategy**. **INITM** scores are significantly lower than any other condition. Significance levels: ^*^p < 0.05, ^***^p < 0.005.

In Table 4 are showed mean and SD of **w** scores for each level of **Strategy**. Multiple comparisons *t*-test using Holm's correction shows that the **w** mean for **INTIM** is significantly lower than all the others (see Figure 6). As consequence, the others conditions are rated as warmer than **INTIM**. **H1ingr**, **H1supp** are thus validated, and **H1intim** and **H2b** are validated for the warmth component.

**Table 4:** Mean and standard deviation of warmth scores for each level of **Strategy**.

**Condition**	**Warmth mean ± SD**
**INGR**	3.77 ± 0.57
**SUPP**	3.54 ± 0.999
**SELF**	3.81 ± 0.70
**INTIM**	2.63 ± 0.93
**RAND**	3.71 ± 0.80
**IMPR**	3.89 ± 0.38

#### 5.6.2. Competence

A 4-way between-subjects ANOVA, including age, sex and Nars scores as factors, was first run in order to check for any effect of these variables. No effects were found for any factor, even when considering only **Strategy** as factor. When looking at the means of **c** for each condition (see Table 5), **SUPP** is the one with lower score, even if its difference with the other scores does not reach statistically significance (all *p*-values > 0.1). **H1supp** and **H1intim** (for the competence component) are not validated.

**Table 5:** Mean and standard deviation of competence scores for each level of **Strategy**.

**Condition**	**Competence mean ± SD**
**INGR**	3.6 ± 0.62
**SUPP**	2.98 ± 0.77
**SELF**	3.75 ± 0.63
**INTIM**	3.65 ± 0.79
**RAND**	3.5 ± 0.70
**IMPR**	3.43 ± 0.76

*No significant differences among the conditions were found*.

#### 5.6.3. User's Perception of the Interaction

We analyzed each item of **perception** separately, by applying non-parametric tests since data were not normally distributed.

**Figure 7:**
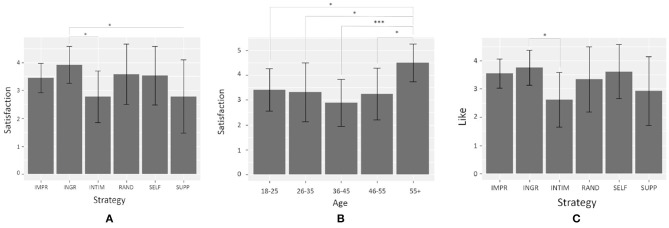
Mean values with sd for the different items of **perception** where an effect of **Strategy** and age was found. Significant results of Dunn's test for multiple comparisons are reported, with the following significance levels: ^*^p < 0.05, ^***^p < 0.001: **(A)** mean values of **satisfaction** for each level of **Strategy**; **(B)** mean values of **satisfaction** for each age range; **(C)** mean values of **like** for each level of **Strategy**.

Concerning **satisfaction** scores, a Kruskal-Wallis rank test showed a statistically significant difference according to **Strategy** [*H*_(5)_ = 11.99, *p* = 0.03]. In particular, Dunn's test for multiple comparisons found that **INGR** scores were significantly higher than **SUPP** (*z* = 2.88, p-adj = 0.03) and **INTIM** (*z* = 2.56, p-adj = 0.04) (see Figure 7A). No differences were found between **IMPR** scores and the other conditions. In addition, a statistically significant difference between scores was found according to Nars scores (*U* = 910.5, *p* = 0.02): participants who got high scores in the Nars questionnaire were more satisfied by the interaction (*M* = 3.62, *SD* = 0.94) than people who got low scores in the Nars questionnaire (*M* = 3.00, *SD* = 1.07). Another interesting results concerns the effect of age on **satisfaction** [*H*(4) = 15.05, *p* = 0.005]: people in the age range 55+ were more satisfied than people of any other age range (see Figure 7B, all p-adj ≤ 0.03).

Concerning **continue** scores, no effect of **Strategy** was found. In general, mean scores were not very high, with only scores in **INGR** and **SELF** conditions being higher than 3. A Mann-Whitney *U*-Test showed a statistically significant difference according to Nars scores (*U* = 998, *p* = 0.001): participants who got high scores in the Nars questionnaire were more motivated to continue the interaction (*M* = 3.28, *SD* = 1.12) than people who got low scores in the Nars questionnaire (*M* = 2.36, *SD* = 1.13).

Concerning **like** scores, a Kruskal-Wallis rank test showed a very near to significance difference according to **Strategy** [*H*_(5)_ = 10.99, *p* = 0.05]. In particular, Dunn's test for multiple comparisons found that **INGR** scores were significantly higher (*M* = 3.75, *SD* = 0.62) than **INTIM** (*M* = 2.62, *SD* = 0.96; *z* = 2.87, p-adj = 0.03) (see Figure 7C). No differences were found between **IMPR** scores and the other conditions. In addition, a statistically significant difference between scores was found according to Nars scores (*U* = 970, *p* = 0.003): participants who got high scores in the Nars questionnaire liked Alice more (*M* = 3.62, *SD* = 0.91) than people who got low scores in the Nars questionnaire (*M* = 2.92, *SD* = 0.99).

Concerning **learnfrom, exhib**, and **rship**, no significant differences in scores were found according to any variable. Participants' scores about **learnfrom** and **exhib** were all over the mean value, while for **rship** the mean scores for each condition were quite low (all means ≤ 2.75), suggesting that participants considered Alice as very distant from them.

Concerning **likeperson** scores, no significant differences were found according to **Strategy**. Mean scores for each condition were quite low (all means ≤ 2.25), suggesting that in general Alice was perceived more similar to a computer than a person. A Mann-Whitney *U*-Test showed a statistically significant difference according to Nars scores (*U* = 1028, *p* = 0.0003): participants who got high scores in the Nars questionnaire perceived Alice less closed to a computer (*M* = 2.49, *SD* = 1.12) than people who got low scores in the Nars questionnaire (*M* = 1.58, *SD* = 0.69).

On the whole, these results do not allow us to validate **H2a**, but agent's adaptation was found to have at least an effect on its level of warmth (**H2b**, see section 5.6.1).

#### 5.6.4. Verbal Cues of Engagement

Only one person gave a negative answer to Topic1_question, while people gave different responses to Topic2_question. In general, participants which did not give much verbal feedback (i.e., <13 reactions over all the speaking turns) gave a positive answer to this question (*OR* = 4.27, *p* = 0.04). In addition, we found that ratings about **likeperson** item were significantly lower for people giving much verbal feedback (*M* = 1, *SD* = 0) compared to those of people who did not talk a lot (*M* = 2.16, *SD* = 1.07; *U* = 36.5, *p* = 0.02). This means that, even than in general users found the agent closer to a computer than to a real person, all the people who gave much verbal feedback during the interaction perceived totally agreed with this definition. No differences in any of the dependent variables were found according to **Strategy**.

## 6. Discussion

In this section we discuss the details of the results of our evaluation study.

First of all, regarding **H1**, the only statistically significant results concern the perception of agent's warmth. Alice was rated as colder when she adopted **INTIM** strategy, compared to the other conditions. This supports the thesis of the primacy of warmth dimension (Wojciszke and Abele, 2008, see section 2), and it is in line with the positive-negative asymmetry effect described by Peeters and Czapinski (1990), who argues that negative information has generally a higher impact in person perception than positive information. In our case, when the agent displays cold (i.e., low warmth) behaviors (i.e., in **INTIM** condition), it is judged by participants with statistically significant lower ratings of warmth. Regarding the other conditions (**INGR**, **SUPP**, **SELF**, **IMPR**, and **RAND**), they elicited warmer impressions in the user, but there is not one strategy better than the others in this regard. The fact that also the **SELF** elicited the same level of warmth than the others could reflect an halo effect: the behaviors displayed to appear competent influenced its warmth perception in the same direction.

Regarding **H2**, the results do not validate our hypothesis **H2a** that the interaction is improved when the virtual agent manages its impressions by adapting its strategy according to user's engagement. When analyzing scores for **perception** items, we found that participants were more satisfied by the interaction and they liked Alice more when the agent wanted to be perceived as warm (i.e., in **INGR** condition), compared to when it wanted to be perceived cold and competent (i.e., in **INTIM** condition). An hypothesis is that since the agent was perceived warmer in **INGR** condition, it could have positively influenced the ratings of the other items, like **satisfaction**. Concerning **H2b** about a possible effect of agent's adaptation on user's perception of its W&C, it is interesting to see that when the agent adapts its self-presentational strategy according to user's overall engagement, it is perceived as warm. This highlights a link between agent's adaptation, user's engagement and warm impression: the more the agent adapts its behaviors, the more the user is engaged and the more s/he perceives the agent as warm.

When looking at participants' verbal cues of engagement (see section 5.6.4), we could divide people into two groups: those who gave much verbal feedback during the speaking turns, and those who mainly answered to agent's questions and did not talk during the rest of the interaction. Participants talking a lot may ask questions to the agent, give their opinion on a game, etc. Since the agent is not endowed with natural language understanding capacities, it could not answer participant's request, nor could it argument on user's opinion. Even though we did not explain agent's limitation to participants before starting the experiment, users who gave many feedback at the beginning of the interaction often became aware that the agent could not react to their speech, since it did not consider what they said, interrupt them, continue talking on its topic as if the participants had not talked. This could had a negative effect on their experience and had led them to choose not to continue to discuss with the agent. When looking at the interaction with this group of people, we notice that they stop proving feedback after the virtual agent missed answering them properly. There is a clear distinction in their verbal behaviors before and after the agent missed their input. In our quantitative analyses we found that the majority of people replying a lot to the agent often gave a negative answer to the question Topic2_question asked by the agent about continuing the discussions. On the other hand, people who did not talk a lot had less probability to experience weird situations such as asking a question to the agent and not being heard. These people were less disappointed than the others and more likely to accept to continue the interaction. Indeed, according to our results, the majority of people who did not give much verbal feedback gave a positive answer to the question Topic2_question. This hypothesis that participants giving much feedback at the beginning of the interaction discovered the limits of the agent seems in line with the lower scores found for **likeperson** item given by people talking a lot compared to the others. The fact that the agent did not behave in the appropriate way and that the agent did not stand up to their expectancies could have highlighted even more the fact that they were in front of a system that simulates a “mock” of interaction. Another possible explanation to this result could concern the fact that people who did not talk a lot were intimidated and so they did not dare to give a negative answer to the agent. This could be in line too with the results about **likeperson** item: considering the agent closer to a person, they could have answered “yes” as not to offend, somehow, the agent.

In this discussion we should take into account how participants' expectancies may affect their perception of the interaction. People expectancies about others' behaviors have already been demonstrated to affect human-human interaction (Burgoon, 1993), as well as when people are in front of an ECA (Burgoon et al., 2016; Biancardi et al., 2018). In this study we found some effects of people's a priori about virtual character: people who got higher scores in the Nars questionnaire generally perceived the agent warmer, compared to people who got lower scores in the Nars questionnaire. In addition, it should not be forgotten that the fact of being in a Sciences museum, combined with people exposition to films and TV shows about artificial intelligence could have had a strong impact on participants' expectancies. People could have difficulties in distinguishing between what is shown in science-fiction films and the current state of the technology of interactive ECAs. Thus, people could have exaggerated expectancies about our virtual agent's capabilities. These expectancies, and the related disappointment showed by some participants when interacting with a less sophisticated virtual character, could have become an uncontrollable variable preventing any other effect of the independent variables of our experiment. Nevertheless, it has to be remembered that in this experiment we mainly focused on the non-verbal behaviors rather than on the dialogical dimension, limiting therefore the dialogue complexity to better control the other variables. The agent had the floor during the majority of the interaction; our system took into account the polarity of user's answers only at 2 specific moments, Topic1_question and Topic2_question (see section 5.5, thus the variability of the agent's dialogue was very limited.

## 7. Conclusions, Limitations, and Future Work

In this paper, we presented a computational model for an Embodied Computational Agent, aimed at managing its self-presentational intentions eliciting different impressions of warmth and competence, in order to maximize user's engagement during the interaction. We built an architecture which takes as input participants facial Action Units, torso and head rotation, use them to compute user's overall engagement and sends it to the dialog manager of the agent. Through a reinforcement learning algorithm which takes user's engagement as reward, the agent can select the self-presentational intention which maximizes user's engagement. In order to evaluate the system, we conceived an interaction scenario where the agent played a role of museum guide. In the experiment we manipulated how the agent selected its self-presentational intention at each speaking turn. It could adapt its behavior by using the reinforcement learning algorithm, or choose it randomly, or use the same self-presentational intention during the whole interaction. The agent which adapted its behavior to maximize user's engagement was perceived as warm by participants, but we did not find any effect of agent's adaptation on users' evaluation of the interaction.

We are aware of some limitations of our system: we discuss them in the following paragraph, and suggest some future improvements to deal with these limitations. First of all, many participants did not like the virtual character, as we can see from their answers to the questionnaires, as well as from their comments during the debriefing. They reported their disappointment about the quality of the animation and of the voice of the agent. They described the experience as “disturbing,” “creepy.” So probably their very first impression about the appearance and the voice of the agent was too strong and affected the rest of the experience. During the interaction, participants did not show many non-verbal behaviors. This could be due to the setup of the experiment, where participants stood in front of the screen and the virtual agent was displayed at human size. According to their comments, many people were a bit frightened by the dimension of the agent and for almost all of them it was their first interaction with an ECA. Many of them stared at the ECA without moving much. They did not vary their facial expression, move their head or gesture. Since our overall engagement detection module relies on the interpretation of non-verbal behaviors, the lack of behavioral change impacts directly the output values it returns.

In our work, we have done qualitative analyses and some quantitative ones. In the future, it would be interesting to conduct further quantitative measures, such as analyzing facial expressions, gaze direction and posture of the participants to measure phenomena like synchronization and alignment. This will allow us to have a complementary measure to their subjective evaluation.

One of the main limits of the interaction was that agent's strategies did not focus on building a rapport with the participant: it just managed its impressions of warmth and competence without considering the social relation with the user. Rapport, meant as the feeling of harmony and connection with another, is an important aspect of human interaction, as well as of human-agent interaction (Gratch et al., 2007; Zhao et al., 2016). Agent's self-presentational intentions should take into account this dimension, at both verbal and non-verbal level. For example, we could include some conversational strategies such as self-disclosure, enhance the gaze behavior of the agent to improve mutual attentiveness, and provide agent's non-verbal listening feedback, such as postural mimicry and synchronization of its movements with the user's ones.

## Data Availability Statement

The datasets generated for this study are available on request to the corresponding author.

## Ethics Statement

This study was exempt from the above requirements, as for the French law, ethical approval is required only for experiments involving invasive interventions on human subjects, which was not the case of this study. All subjects gave written informed consent in accordance with the Declaration of Helsinki. The protocol followed the ethical guidelines of the Institut des Systémes Intelligents et de Robotique.

## Author Contributions

BB wrote the manuscript with support and feedback from all the other authors. More specifically, MM collaborated to the implementation of the system and was in charge of the system architecture section. CP globally supervised the manuscript. PL was the main contributor of the design, implementation of the system, and collaborated to run the experiment.

### Conflict of Interest

The authors declare that the research was conducted in the absence of any commercial or financial relationships that could be construed as a potential conflict of interest.

## Footnotes

^1^https://www.microsoft.com/en-us/download/details.aspx?id=27225
